# Sensitive SYBR Green—Real Time PCR for the Detection and Quantitation of Avian Rotavirus A

**DOI:** 10.3390/vetsci6010002

**Published:** 2018-12-29

**Authors:** David De la Torre, Claudete S. Astolfi-Ferreira, Ruy D. Chacon, Antonio J. Piantino Ferreira

**Affiliations:** Department of Pathology, School of Veterinary Medicine, University of São Paulo, São Paulo 05508-270, Brazil; daviddelatorreduque@gmail.com (D.D.l.T.); csastolfi@gmail.com (C.S.A.-F.); ruychaconv@usp.br (R.D.C.)

**Keywords:** avian rotavirus, real-time PCR, RT-qPCR, SYBR Green

## Abstract

Avian rotavirus A (ARtV-A) is a virus that affects young birds, causing acute diarrhea and economic losses in the poultry industry worldwide. The techniques used for the diagnosis of ARtV-A include electron microscopy, isolation in cell culture, and serology, as well as molecular techniques, such as the reverse transcription-polymerase chain reaction (RT-PCR). The objective of this work was to standardize a real-time RT-polymerase chain reaction (RT-qPCR) using SYBR Green chemistry for the rapid detection and quantification of ARtV-A from bird tissues and materials fixed on FTA cards on the basis of the nucleotide sequence of segment 6 (S6), which codes for the structural VP6 protein of ARtV-A. The results show the efficient amplification of the proposed target, with a limit of detection (LoD) of one copy gene (CG) per microliter of cDNA and a limit of quantification (LoQ) of 10 CGs per microliter. The efficiency of the primers was determined to be 95.66% using a standard curve, with an R^2^ value of 0.999 and a slope of −3.43. The specificity was determined using samples coinfected with ARtV-A, the chicken parvovirus, the chicken astrovirus, and the avian nephritis virus as positive controls and commercially available vaccines of the infectious bronchitis virus, infectious bursa disease virus, avian reovirus and healthy organs as negative controls. This technique, which lacks nonspecific PCR products and dimers, demonstrated greater sensitivity and specificity than conventional RT-PCR, and it reduced the analysis time by more than 50%.

## 1. Introduction

The rotavirus is one of the etiological agents most closely associated with enteric problems in young animals, including both mammals and birds [1,2,3]. The avian rotavirus A (ARtV-A) is a highly contagious virus prevalent around the world; ARtV-A mainly affects young birds, causing acute diarrhea and decreasing the zootechnical performance of commercial birds, which causes an economic impact on the poultry industry [4,5]. ARtV-A has also been encountered in commercial birds with growth retardation, runting-stunting syndrome and variable mortality; ARtV-A is commonly associated with other enteric viruses such as the chicken parvovirus (ChPV), the chicken astrovirus (CAstV), the avian nephritis virus (ANV) and the avian reovirus (AReo) [6,7,8,9,10]. The genus *Rotavirus* belongs to the Reoviridae family. The rotavirus is a non-enveloped virus with a segmented dsRNA genome. The 11 segments of the rotavirus genome code for six structural proteins (VP1, VP2, VP3, VP4, VP6, and VP7) and five nonstructural proteins (NSP1, NSP2, NSP3, NSP4 and NSP5/NSP6) [11]. The classification of rotaviruses is determined by the organization of the genome, the structure of the capsid and the viral replication strategy. Rotavirus serogroups are classified by the antigenic structure of the intermediate capsid, VP6, and they are designated by the letters A to H [12]. Only serogroups A, D, F and G have been associated with infection in birds [13,14]. The objective of this work is to standardize a real-time RT-polymerase chain reaction (RT-qPCR) for the rapid detection and quantification of ARtV-A based on a nucleotide fragment of the genome in segment 6 (S6) that codes for the VP6 protein.

## 2. Materials and Methods

### 2.1. Sampling

We used twenty-four samples of commercial poultry organs stored in the Laboratory of Avian Diseases at the School of Veterinary Medicine—University of São Paulo. These samples were obtained through the Avian Disease Diagnostic Service between the years 2013 and 2016. Each sample was collected from chickens exhibiting diarrhea, delayed growth, and other signs associated with the runting-stunting syndrome. A total of 20/24 samples were collected from Brazilian poultry farms, 18/24 of these samples were previously diagnosed with ARtV-A and other enteric viruses such as ChPV, ANV, CAstV and infectious bronchitis virus (IBV) with conventional PCR [9,15], and 2/24 were randomly chosen from young birds that had acute diarrhea and growth retardation. The remaining 4/24 samples were collected from commercial poultry from Ecuador, which were transported using FTA cards for viral diagnosis.

### 2.2. RNA Extraction

The samples (stored at −20 °C) were thawed on ice, and a portion of each sample was transferred to a 1.5 mL microfuge tube with 750 μL of phosphate buffered saline pH 7.2 at a 1:1 ratio. The suspension was homogenized and subjected to three cycles of freezing and thawing to disrupt cellular membranes. The suspension was then clarified by centrifugation at 12,000× *g* for 30 min at 4 °C. A 250 μL aliquot of the supernatant was used for RNA extraction. RNA extraction was performed by phenol-chloroform extraction according to the protocol described by Tony et al. [16]. The concentration of RNA extracted from each sample was measured using a NanoDrop ONE (Thermo Fisher Scientific, Wilmington, DE, USA) and then immediately subjected to reverse transcription reaction.

### 2.3. Primer Design

**Figure 1:**
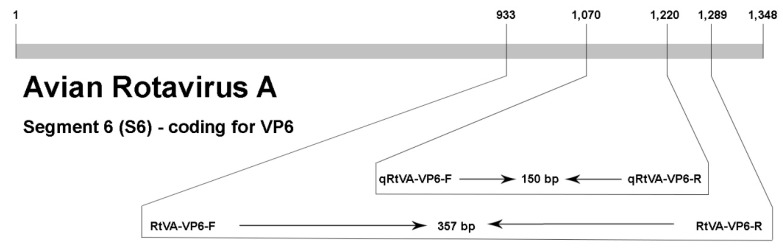
Diagram of segment S6 of ARtV-A, indicating the position of the primers used for RT-PCR and RT-qPCR within the nucleotide sequence. F: forward, R: reverse, bp: base pair.

Two pairs of primers were designed for this study from a highly conserved region of segment 6 (S6) that codes for the intermediate capsid (VP6) of the ARtV-A. Forty-seven nucleotide sequences of S6 of ARtV-A from GenBank were aligned using the CLUSTAL W method available in Clustal X 2.0 software (Des Higgins Conway Institute, UCD, Dublin, Ireland) to determine the variable and conserved regions within this genome segment. The aligned nucleotide sequences were from strains isolated from Nigeria, Germany, South Korea, and Brazil. Primers were designed using Primer 3 Plus software [17]. One pair of primers (RtVA-VP6-F and RtVA-VP6-R, Table 1) was designed to be used for conventional RT-PCR. These primers produced a DNA fragment of 357 bp, which was used as a template for the construction of the standard curve in RT-qPCR. The second pair of primers (qRtVA-VP6-F and qRtVA-VP6-R, Table 1) was designed as an internal primer, spanning a fragment of 150 bp, and these primers were used for RT-qPCR. A diagram of segment 6, with the position of the primers indicated, is shown in Figure 1. Random primers, Oligo (dT)_12_ primers, and the two sets of primers for RT-PCR and RT-qPCR were synthetized by Life Technologies (São Paulo, Brazil).

### 2.4. Reverse-Transcription Reaction

The reverse transcription reaction was performed using 3 µL of the extracted RNA, which contained between 1 ng to 5 × μg total RNA. The RNA was denatured at 95 °C for 5 min, chilled on ice, and then added to a mixture containing 250 ng of random primers, 10 mM of each deoxynucleotide triphosphate (dNTP), 4 μL of 5 primer buffer (250 mM Tris-HCl pH 8.3, 375 mM KCl, 15 mM Magnesium Chloride) (Invitrogen, Carlsbad, CA, USA), 2 μL of 100 mM dithiothreitol (DTT) (Invitrogen, Carlsbad, CA, USA), 200 units of Moloney murine leukemia virus reverse transcriptase (M-MLV RT)(Invitrogen, Carlsbad, CA, USA), and Ultrapure™, RNase and DNase free water (Invitrogen, Grand Island, NY, USA) to reach a total volume of 20 μL per reaction. Thermal cycles were programed according to the M-MLV manufacturer’s instructions.

### 2.5. Reverse-Transcription PCR (RT-PCR)

One sample that was ARtV-A positive was subjected to RT-PCR using 2.5 µL of the previously synthetized cDNA as a template. The cDNA was added to a mixture containing 1 × magnesium free (-Mg) PCR Buffer (Invitrogen), 1.25 mM of each deoxynucleotide triphosphate, 0.6 µM of each primer (RtVA-VP6-F and RtVA-VP6-R, Table 1), 0.5 U of Platinum™ Taq DNA Polymerase, 1.5 mM of MgCl_2_ (Invitrogen, Carlsbad, CA, USA), and Ultrapure™, RNase and DNase free water (Invitrogen, Carlsbad, CA, USA), to reach a total reaction volume of 25 µL. The following cycling conditions were used for the reaction in a ProFlex™ PCR System (Applied Biosystems, Marsiling, Singapore): One cycle of 94 °C for 5 min, 35 cycles at 94 °C for 60 s, 60 °C for 45 s, and 72 °C for 1 min, followed by a final extension at 72 °C for 10 min. The presence of a positive RT-PCR product was confirmed by gel electrophoresis in a 1.5% agarose gel. DNA was stained with SYBR^®^ Safe DNA gel stain (Invitrogen, Carlsbad, CA, USA), and compared with a 100 bp DNA Ladder (Invitrogen). The size of the DNA band was analyzed in an UV transilluminator and photographed using an Alpha Imager Mini Analysis System (Alpha Innotech-ProteinSimple, San Jose, CA, USA). 

### 2.6. Reverse Transcription qPCR (RT-qPCR)

All samples were subjected to RT-qPCR in triplicate to detect and quantify the number of copies of genes (CGs) of ARtV-A present in the previously synthetized cDNA. Each reaction used 1 μL of cDNA, 0.6 μM of each primer, (qRtVA-VP6-F and qRtVA-VP6-R, Table 1), 5 μL of PowerUp™ SYBR® Green Master Mix (Applied Biosystems, Austin, TX, USA) and Ultrapure™, RNase and DNase free water (Invitrogen, Carlsbad, CA, USA) to reach a total reaction volume of 10 μL. The number of cycles and the cycling times were configured using the FAST method of the QuantStudio3 Real Time PCR System (Applied Biosystems, Marsiling, Singapore) according to the PowerUp™ SYBR® Green Master Mix (Applied Biosystems, Austin, TX, USA) manufacturer’s instructions as follows: One cycle at 50 °C for 2 min for activation of the Uracil-DNA glycosylase enzyme, one cycle at 95 °C for 2 min, and then 40 cycles at 95 °C for 1 s for template denaturation and 60 °C for 30 s for annealing and extension. To plot the dissociation curve (melting curve), the following conditions were used: 95 °C for 15 s, 60 °C for one minute and 95 °C for 15 s, with a gradual increase of temperature of 1.6 °C/s between the first and second stages, and 0.15 °C/s to reach the final stage. 

### 2.7. Standard Curve Construction

A standard curve was constructed using the RT-PCR product described above. The product was purified using the GPX ™ PCR DNA and Gel Band Purification kit (GE Healthcare, Piscataway, NJ, USA) according to the manufacturer’s instructions. The purified DNA was quantified with the Qubit™ 4 fluorometer (Life Technologies Pte Ltd., Singapore), and the number of CGs was calculated with the DNA Copy Number and Dilution Calculator tool available at www.thermofisher.com. The DNA was adjusted to a starting concentration of 1E9 CG/µL, and ten serial dilutions were created at a dilution factor of 1:10. This standard curve was used to determine the sensitivity and amplification efficiency of the RT-qPCR reaction.

### 2.8. Analytical Sensitivity and Specificity 

To determine the specificity of the RT-qPCR, this reaction was run using vaccine samples from other concomitant viruses such as avian infectious bronchitis virus (IBV), avian reovirus (AReo), infectious bursa disease (IBD) and five healthy organ samples. The limit of detection (LoD) was estimated by the lowest RT-PCR product concentration that could be amplified by RT-qPCR, and the limit of quantification (LoQ) was estimated by the lowest RT-PCR product concentration that the assay could quantify by RT-qPCR and were maintained within the linear range of the standard curve.

### 2.9. DNA Sequencing and Data Analysis

The PCR product from the RT-PCR reaction was purified using the GPX™ PCR DNA and Gel Band Purification kit (GE Healthcare, Piscataway, NJ, USA) according to the manufacturer’s instructions. The purified DNA was sequenced in both the forward and reverse directions using the BigDye® Terminator Cycle Sequencing Kit v3.1 (Invitrogen, Carlsbad, CA, USA) according to the manufacturer’s instructions. The sequencing product was analyzed with an ABI 3730 DNA Analyzer (Applied Biosystems, Foster City, CA, USA). The nucleotide sequences obtained were edited on the CLC Main Workbench 7.7.3 (CLC Bio-Qiagen, Aarhus, Denmark) and aligned to the 47 sequences used for the primer design as described above. 

## 3. Results

### 3.1. RT-qPCR

**Figure 2:**
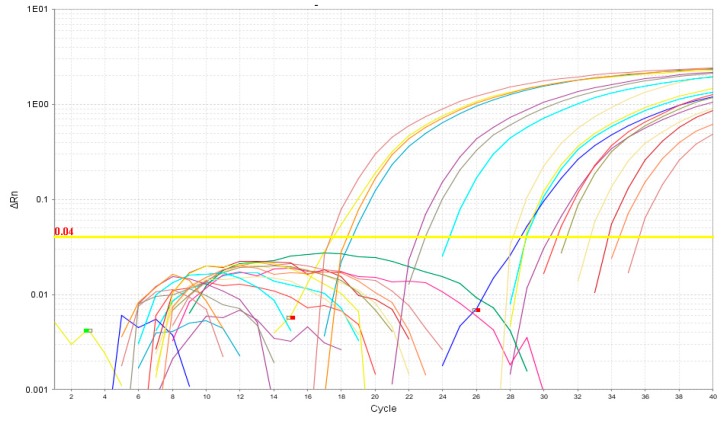
Logarithmic amplification plot.

**Figure 3:**
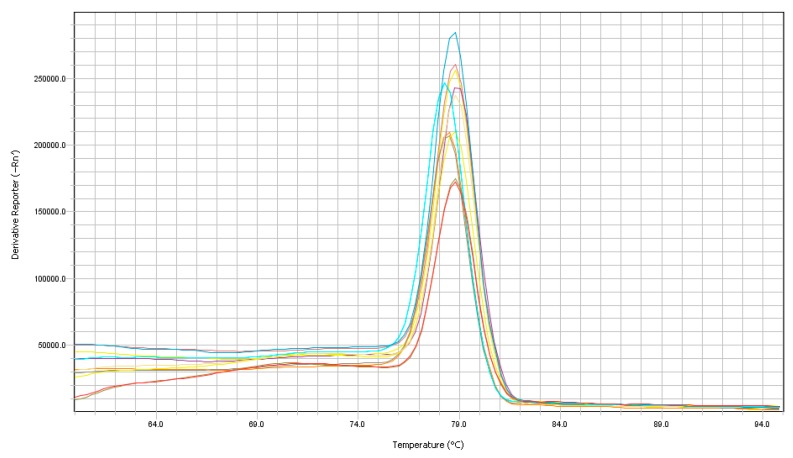
Melt curve plot.

All 24 samples were amplified by RT-qPCR. The samples did not show alterations in the linear or logarithmic amplification curves, and no dimers were amplified in any reaction, demonstrating a successful design and good concentration of the primers used (Figure 2 and Figure 3). A positive control corresponding to the 1 × 10^5^ dilution from the standard curve was used for the validation of the dissociation curve, which demonstrated that a similar sequence was amplified in all the samples tested with an average Tm of 78.4 °C ± 0.46 in the highest peak. The running time for the qPCR reaction was 45 min using the Fast mode, which was compatible with the kit used in this experiment.

### 3.2. Standard Curve

**Figure 4:**
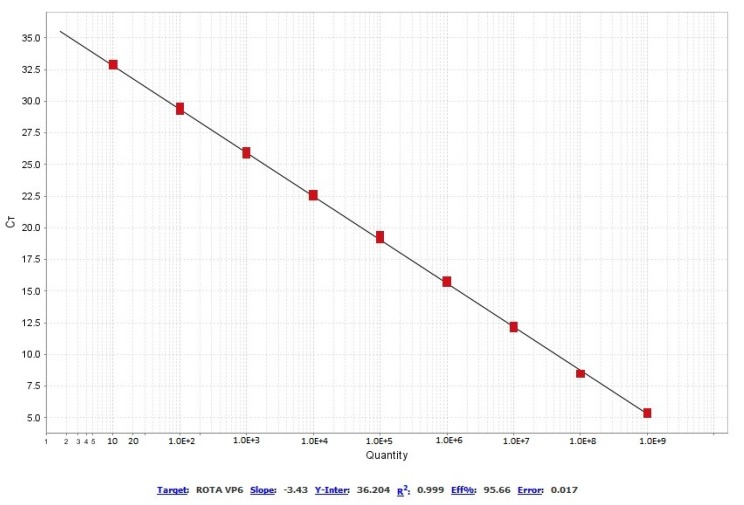
Standard curve diagram.

The serial dilutions were amplified as expected, with Ct values increasing at a proportional rate between each of them. The efficiency percentage of the primers was 95.66% and the coefficient of determination (R^2^) was 0.999. The slope of the standard curve was −3.43, and the Y-intercept was 36.204 (Figure 4). The reproducibility of the intra-assay RT-PCR results from triplicate reactions of nine serial dilutions are shown in Table 2. The coefficients of variation were relatively low (< 1.69%), ranging between 0.30% and 1.69%.

### 3.3. Analytical Sensitivity and Specificity 

The limit of detection (LoD) was 1 CG per μL of purified RT-PCR product. The limit of quantification (LoQ) was determined in 10 CG per μL of purified RT-PCR product, given that the Ct value of the dilution with 1 viral particle per μL was outside the linear range of the standard curve. No amplification was observed in samples from healthy birds or other pathogen vaccine samples.

## 4. Discussion

ARtV-A is a prevalent virus throughout the world, and it has been associated with enteric problems in young chickens, with an economic impact in the poultry industry; ARtV-A causes diarrhea and growth retardation, and it is associated with runting-stunting syndrome [2,10,18]. The main methods to prevent the transmission of the virus include proper management of biosecurity of farms and early diagnosis of the virus in birds [7]. Cell culture isolation, serological methods, and electron microscopy have been the most widely used techniques for the diagnosis of rotavirus in all species [19]. Currently, RT-PCR is used as a molecular diagnostic method, which allows for the identification of the presence of ARtV genomes with a high degree of sensitivity [20,21]. However, this technique involves very delayed procedures in addition to the extraction of genetic material and the synthesis of cDNA molecules from viral RNA. In our work using RT-qPCR, we achieved a diagnostic response in just 45 min compared to the 2 h necessary for conventional RT-PCR. Additionally, conventional PCR product requires testing on an agarose gel and would require an extra time of approximately 90 min [15]. In addition to the time, the sensitivity of RT-qPCR is greater than that of conventional RT-PCR; in our test, we were able to detect one gen copy per µL of cDNA (LoD) compared to the LoD (10 CG) by conventional RT-PCR developed by Day et al. [15], which targets the NSP4 gen. This increased sensitivity of RT-qPCR was also demonstrated for other rotavirus species [22,23] and was demonstrated by Mo et al. [24], who conducted a study that compared the detection between RT-PCR and RT-qPCR. An additional and very important advantage of RT-qPCR is that absolute quantifications of the CG number of the virus can be calculated, which is not possible with conventional RT-PCR. The efficiency of the reaction is also reflected by the absence of dimers and non-specific products; by the presence of a single peak in the dissociation curve (Figure 3); in the efficiency indices (95.6%) and coefficient of determination R^2^ (0.999) of the standard curve (Figure 4). These values indicate the high sensitivity of our RT-qPCR reaction, both for samples from organic tissues (spleen, pancreas and intestines) and for samples preserved on FTA cards, demonstrating the overall suitability of the designed primers [25]. Additionally, RT-qPCR products were genetically sequenced and showed identity with ARtV-A strains in GenBank. ARtV-A was detected in samples from young birds showing signs of runting-stunting syndrome that were infected with multiple viruses such as the chicken parvovirus, the chicken astrovirus, and the avian nephritis virus, demonstrating that the amplification of ARtV-A is not affected by the presence of other viral pathogens. Alternatively, no amplification was observed in the vaccine samples from other viruses that affect poultry, such as IBV, IBD, and AReo. Random primers were used for reverse transcription to detect other potential RNA viruses that could interfere with ARtV-A screening [23]. Our RT-qPCR reaction can detect and quantify ARtV-A; however, the characterization of the viral genotype would require analysis of the genes that code for VP4 and VP7, which are currently analyzed by Sanger or next-generation sequencing (NGS) [26,27].

## 5. Conclusions 

Our proposed method of RT-qPCR based on SYBR Green chemistry showed the rapid and efficient detection and quantification of ARtV-A. The LoD and LoQ allow this procedure to be used for the diagnosis of ARtV in bird tissues such as the pancreas, intestines, or spleen, as well as in fixed material on FTA cards. These methods increase the accuracy of positive and negative results and reduce processing time compared with conventional techniques such as cell culture, electron microscopy, and conventional RT-PCR. Subsequent works applied with this proposed method, may contribute to the complete validation of our assay.

## Figures and Tables

**Table 1:** Primers designed for this study and their location within the Segment 6 of ARtV-A.

Name	Gene Target	Assay	Sequence	Nt Position	Reference
RtVA-VP6-F	VP6	RT-PCR	5′-GTAGCAGCACTTTTCCCAGT-3′	933-953	This study
RtVA-VP6-R			5′-GTCCGCTACGGACTATTCG-3′	1289-1271	
qRtVA-VP6-F	VP6	RT-qPCR	5′-TTGGACCAGTATTTCCTGCTG-3′	1070-1091	This study
qRtVA-VP6-R			5′-TGGTATGAGCTGTTACCCTCAA-3′	1220-1198	

F: forward, R: reverse, nt: nucleotide.

**Table 2:** Reproducibility of the RT-qPCR assay used for the detection of the VP6 gene of ARtV-A.

Concentration of the RT-PCR Product (DNA copies/µL)	Ct Mean	Ct SD	%CV
1.0 × 10^1^	32.62	0.51	1.57%
1.0 × 10^2^	29.35	0.16	0.55%
1.0 × 10^3^	25.90	0.15	0.59%
1.0 × 10^4^	22.57	0.13	0.56%
1.0 × 10^5^	19.23	0.17	0.90%
1.0 × 10^6^	15.74	0.10	0.64%
1.0 × 10^7^	12.21	0.11	0.93%
1.0 × 10^8^	8.47	0.03	0.30%
1.0 × 10^9^	5.37	0.09	1.69%

Ct: cycle treshold, SD: standard deviation, CV: coefficient variation.
